# Magnetic Core–Shell Molecularly Imprinted Nano-Conjugates for Extraction of Antazoline and Hydroxyantazoline from Human Plasma—Material Characterization, Theoretical Analysis and Pharmacokinetics

**DOI:** 10.3390/ijms22073665

**Published:** 2021-04-01

**Authors:** Joanna Giebułtowicz, Natalia Korytowska, Monika Sobiech, Sebastian Polak, Barbara Wiśniowska, Roman Piotrowski, Piotr Kułakowski, Piotr Luliński

**Affiliations:** 1 Department of Bioanalysis and Drugs Analysis, Faculty of Pharmacy, Medical University of Warsaw, Banacha 1, 02-097 Warsaw, Poland; natalia.korytowska@wum.edu.pl; 2 Department of Organic Chemistry, Faculty of Pharmacy, Medical University of Warsaw, Banacha 1, 02-097 Warsaw, Poland; monika.sobiech@wum.edu.pl (M.S.); piotr.lulinski@wum.edu.pl (P.L.); 3 Department of Social Pharmacy, Faculty of Pharmacy, Jagiellonian University Medical College, Medyczna 9, 30-688 Kraków, Poland; sebastian.polak@uj.edu.pl (S.P.); b.wisniowska@uj.edu.pl (B.W.); 4 Department of Cardiology, Postgraduate Medical School, Grochowski Hospital, 04-073 Warsaw, Poland; rpiotrow@op.pl (R.P.); kulak@kkcmkp.pl (P.K.)

**Keywords:** antazoline, atrial fibrillation, biomedical applications, core–shell nanostructures, dispersive solid phase extraction, hydroxyantazoline, magnetic molecularly imprinted polymers, mass spectrometry, particle characterization

## Abstract

The aim of this study was to develop magnetic molecularly imprinted nano-conjugate sorbent for effective dispersive solid phase extraction of antazoline (ANT) and its metabolite, hydroxyantazoline (ANT-OH) in analytical method employing liquid chromatography coupled with mass spectrometry method. The core–shell material was characterized in terms of adsorption properties, morphology and structure. The heterogeneous population of adsorption sites towards ANT-OH was characterized by two K_d_ and two B_max_ values: K_d_ (1) = 0.319 µg L^−1^ and B_max_ (1) = 0.240 μg g^−1^, and K_d_ (2) = 34.6 µg L^−1^ and B_max_ (2) = 5.82 μg g^−1^. The elemental composition of magnetic sorbent was as follows: 17.55, 37.33, 9.14, 34.94 wt% for Si, C, Fe and O, respectively. The extraction protocol was optimized, and the obtained results were explained using theoretical analysis. Finally, the analytical method was validated prior to application to pharmacokinetic study in which the ANT was administrated intravenously to three healthy volunteers. The results prove that the novel sorbent could be useful in extraction of ANT and ANT-OH from human plasma and that the analytical strategy could be a versatile tool to explain a potential and pharmacological activity of ANT and ANT-OH.

## 1. Introduction

In recent years, many advances have been made in the field of biomedical and food analysis mainly due to developments of hyphenated analytical techniques like liquid chromatography coupled with mass spectrometry (LC-MS). However, biological matrices contain diverse components with different chemical properties, which greatly hinder the selective extraction of target analytes, reducing the sensitivity of the instrumental analytical methods. Therefore, sample pretreatment methods are constantly developing aiming to reduce the matrix effect and improve the method selectivity. One of the most effective method is solid phase extraction (SPE), reliable and cost-effective technique for the selective isolation and concentration of a wide range of analytes and sample matrices [1]. However, low selectivity of commercial sorbents used in the pretreatment step limited the applicability of the technique. Here, the molecularly imprinted polymers (MIPs) could be recognized as attractive sorbents. The MIPs are characterized by satisfactory selectivity derived from template-tailored synthesis. Currently, those materials found application as sorbents in the separation processes or recognition elements of detection devices [2,3,4,5].

There are various modifications and iterations of SPE including solid phase microextraction, stir bar sorptive extraction, matrix solid phase dispersion, ion-exchange solid phase extraction and dispersive solid phase extraction (d-SPE) [6]. The d-SPE is one of the most approachable iterations of SPE that ensures high contact surface between sorbent and sample, allowing for the extraction equilibrium to be reached quickly [6]. The resulting method is proven to be effective and rapid, especially if performed on magnetic particles that provide quick extraction of the target compound, as they can be readily recovered by a magnet [7].

Determination of pharmaceuticals and their metabolites in biological fluids is an essential and important part of the drug development process and therapeutic drug monitoring. Antazoline (N-benzyl-N-(4,5-dihydro-1H-imidazo-2-ylmethyl)aniline, ANT) is a very effective drug in rapid conversion of recent-onset atrial fibrillation (AF) to sinus rhythm [8,9,10]. ANT prolonged P wave, QRS duration and QT/QTc, which corresponded with drug-induced prolongation of conduction (P wave and QRS) and repolarization (QT/QTc) [11], increasing sinus rhythm and prolonged interatrial conduction [12]. However, the insufficiency of data on ANT properties together with only one randomized clinical trial described in the literature could explain the reason that ANT is not listed in the formal guidelines [13]. Nevertheless, recent data on pharmacokinetics presenting fast elimination and relative high volume of distribution of the drug [14]. So far fifteen Phase I and Phase II metabolites of ANT were described with four of them being Phase I metabolites. The most prominent Phase I metabolites of ANT are N-benzyl-1-(4,5-dihydro-1H-imidazol-2-yl)methanamine and hydroxyantazoline (4-(N-benzyl-N-(4,5-dihydro-1H-imidazol-2-yl)methyl)aminophenol, ANT-OH) (semi quantitative analysis) [15]. However, lack of data on pharmacological activity, toxicity and pharmacokinetics of the metabolites hampers better understanding of the activity profile of ANT and its potential clinical application. It is well known that the pharmacological activities of metabolites, comparing to parent drug, could vary significantly. The examples of hydroxymetronidazole or hydroxybuproprion proved that metabolites can be less active than the parent drug. In contrary, S-hydroxyrisperidone or 4-hydroxyatorvastatin are examples of more potent metabolites of their parent drugs [16]. Since ANT-OH is one of the most important ANT metabolites with similar structure to ANT, it could be supposed that the compound reveals similar antiarrhythmic effect. Thus, to reveal the data of ANT-OH pharmacokinetics, a new analytical method capable for determination of low concentration of analytes in the complex sample should be proposed.

So far there are a few methods of ANT determination which are based on liquid-liquid extraction (LLE) or cloud-point extraction (CPE) [14,17,18,19]. It should be underlined that the LLE is an effective and fast extraction method, but requires the use of organic solvents which are toxic to laboratory staff as well as expensive to dispose of, and in CPE various surfactants are used instead of organic solvent to make the method safer and more environment-friendly [1]. However, until now no analytical method for the determination of ANT-OH or simultaneous analysis of ANT and ANT-OH in human plasma has been proposed.

The aim of the study was to develop magnetic molecularly imprinted sorbent to be used in pretreatment of plasma samples prior to LC-MS analysis for simultaneous determination of ANT and its metabolite, ANT-OH, in human plasma. The ANT and ANT-OH were chosen as model compounds to verify the extraction applicability of newly synthesized molecularly imprinted nano-conjugates. The analytes were extracted using d-SPE and core–shell nano-conjugates of MIPs and magnetic material to facilitate and speed-up the process. The sorbent was synthesized and characterized in terms of adsorption properties, morphology and structure. The separation protocol of magnetic d-SPE was optimized and the analytical method was validated prior to application to pharmacokinetic study of ANT and ANT-OH after intravenous administration to healthy volunteers of 100 mg of antazoline mesylate.

## 2. Results and Discussion

### 2.1. Characterization of Magnetic Sorbent

#### 2.1.1. Adsorption Properties

In order to reveal the adsorption characteristics of mag-MIP (magnetic molecularly imprinted polymer) and mag-NIP (magnetic non-imprinted polymer), the analysis of binding capacities was calculated according to Equation (1) (in a range of low concentrations of ANT and ANT-OH). The adsorption properties were evaluated using Freundlich model, Equation (2).

**Figure 1:**
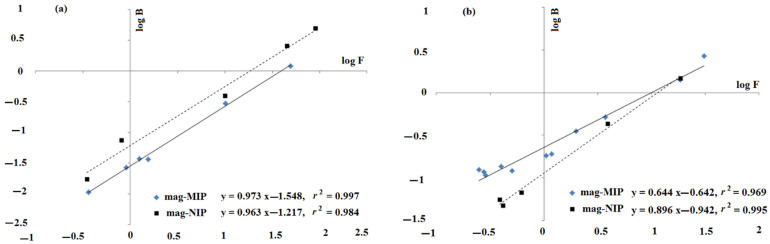
Freundlich isotherms for ANT (**a**) and ANT-OH (**b**) on mag-MIP and mag-NIP.

The Freundlich model, the most popular adsorption model for a single solute system, is based on the distribution of solute between the solid phase and aqueous phase at equilibrium. The model is valid for heterogeneous surfaces and it implies that the adsorption energy exponentially decreases on the finishing point of the adsorption regions of an adsorbent. The straight lines of log *B* versus log *F* for ANT and ANT-OH on mag-MIP and mag-NIP were characterized by the regression coefficients of *r*^2^ > 0.984, confirming that the adsorption could be described by the Freundlich equation (Figure 1). The estimated values of the heterogeneity indices, *m* for ANT on mag-MIP and mag-NIP, were 0.97 and 0.96, respectively. The results revealed homogeneous population of adsorption sites of both sorbents towards ANT. However, the analysis of heterogeneity indices for ANT-OH confirmed heterogeneous population of adsorption sites on mag-MIP, in contrast to mag-NIP (*m* value of 0.64 and 0.90, respectively). The heterogeneity increased as the value of *m* decreased. Thus, to characterize the adsorption of ANT-OH on mag-MIP and mag-NIP more comprehensively, the Langmuir model transformed to Scatchard equation (Equation (3)) was employed.

**Figure 2:**
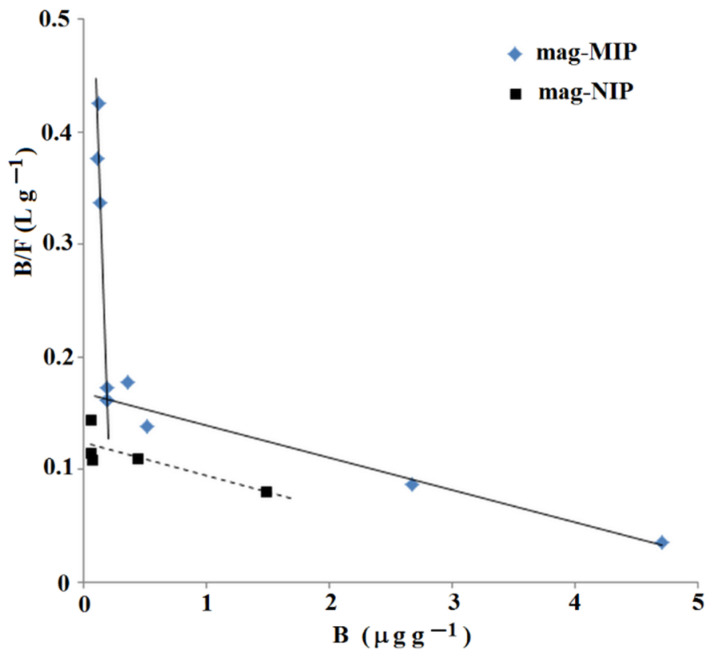
Scatchard plot for ANT-OH on mag-MIP and mag-NIP.

The system that has more than one population of adsorption sites is characterized by more than one line. The Scatchard plot for ANT-OH on mag-MIP and mag-NIP is presented in Figure 2. The analysis revealed two straight lines for mag-MIP and only one line for mag-NIP. It means that two classes of adsorption sites for ANT-OH were predominant in mag-MIP with two *K*_d_ and two *B*_max_ values: *K*_d_ (1) = 0.319 µg L^−1^ and *B*_max_ (1) = 0.240 μg g^−1^ for the higher affinity adsorption sites, and *K*_d_ (2) = 34.6 µg L^−1^ and *B*_max_ (2) = 5.82 μg g^−1^ for the lower affinity adsorption sites. Thus, the mag-MIP possessed specific adsorption sites for ANT-OH. In contrast, the mag-NIP had only one low affinity adsorption site characterized by the value of *K*_d_ = 33.8 µg L^−1^ and *B*_max_ = 4.19 μg g^−1^.

**Figure 3:**
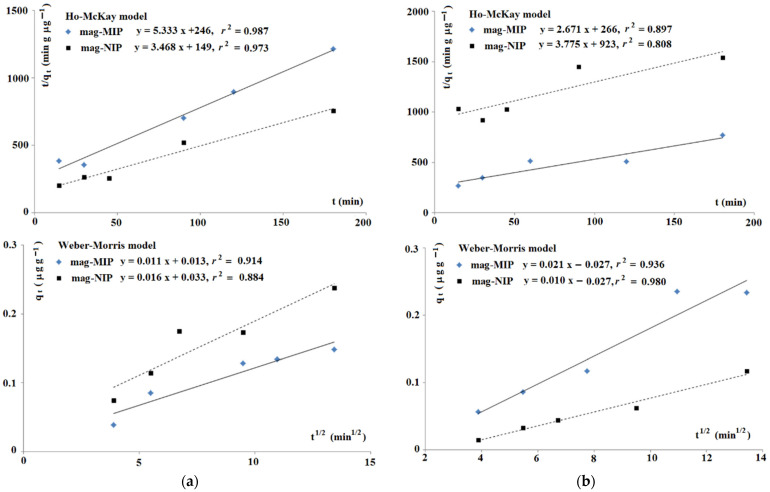
Kinetics data on mag-MIP and mag-NIP for ANT (**a**) and ANT-OH (**b**).

Next, the adsorption kinetics of ANT and ANT-OH on mag-MIP and mag-NIP were evaluated because the time of adsorption is an important parameter during the optimization of d-SPE. Obtained kinetics data on mag-MIP and mag-NIP for ANT and ANT-OH fitted well to Ho-McKay and Weber-Morris models (Figure 3).

First, the data fitted into the model of Ho-McKay were analyzed [20]. This model could be used universally to various systems governed by different mechanisms, but is limited because did not identify a rate-controlling mechanism. The adsorption of ANT and ANT-OH on both materials gave a linear function *t*/*q* against *t*. The linearities closer to one were obtained for ANT data. The calculated values *k*_2_ and *q*_e_ were as follows; for ANT, mag-MIP: *k*_2_ = 0.116 g μg^−1^ min^−1^, *q*_e_ = 0.188 µg g^−1^, and for mag-NIP: *k*_2_ = 0.081 g μg^−1^ min^−1^, *q*_e_ = 0.288 µg g^−1^, as well as for ANT-OH, mag-MIP: *k*_2_ = 0.027 g μg^−1^ min^−1^, *q*_e_ = 0.374 µg g^−1^, and for mag-NIP: *k*_2_ = 0.015 g μg^−1^ min^−1^, *q*_e_ = 0.265 µg g^−1^. To extend the analysis, the Weber-Morris model was employed [21]. This model, which is based on Fick’s second law of mass transfer, is widely used to describe the mass transfer mechanism from the outer surface to pores of adsorbent during the sorption process. The calculated values *k*_3_ were as follows; for ANT, mag-MIP: 0.016 µg g^−1^ min^−1/2^, and for mag-NIP: 0.011 µg g^−1^ min^−1/2^ as well as for ANT-OH, mag-MIP: 0.021 µg g^−1^ min^−1/2^, and for mag-NIP: 0.010 µg g^−1^ min^−1/2^. The non-linear characteristics for both compounds on mag-MIP and non-zero point-crossing of origin suggests that the sorption mechanism is more complex. It could indicate that the intra-particles diffusion is not the only rate-controlling step.

#### 2.1.2. Theoretical Insight into Adsorption Process

The theoretical analysis is a powerful tool in the prediction of adsorption behavior of MIPs. It was utilized to create the models of the MIPs cavity in order to estimate the selectivity of the sorbent [22,23,24,25,26]. In order to provide insight into the mechanism that governs the adsorption of ANT and ANT-OH on the MIP, the analysis of interactions of chosen analytes: ANT or ANT-OH in different forms: neutral (ANT, ANT-OH), cationic (ANT^+^), anionic (ANT-O^–^) or zwitterionic (ANT^+^-O^–^) in the MIP cavity were performed, mimicking the process of adsorption of target analyte inside the cavity after template removal. Five models of systems consisting of polymer cavity, solvent and analyte were studied according to different structures of analytes: ANT, ANT^+^_,_ ANT-OH, ANT-O^–^, ANT^+^-O^–^. The values of Δ*E_B_* for respective systems were as follows (in kcal mol^−1^): –57.0 (ANT), +97.7 (ANT^+^), +97.8 (ANT-OH), –92.5 (ANT-O^–^) and +73.7 (ANT^+^-O^–^).

As it could be seen, the only negative values of Δ*E_B_* were obtained for models of ANT and ANT-O^–^. The negative values of Δ*E_B_* proved good stability of those systems and positive processing of adsorption. The results could also suggest that in the conditions of experimental and computational analysis, the most important role was played by the neutral form of ANT and anionic form of ANT-O^–^. Thus, those two systems were selected further for the comprehensive analysis of interactions between the analyte (ANT or ANT-O^–^) and the MIP. 

**Figure 4:**
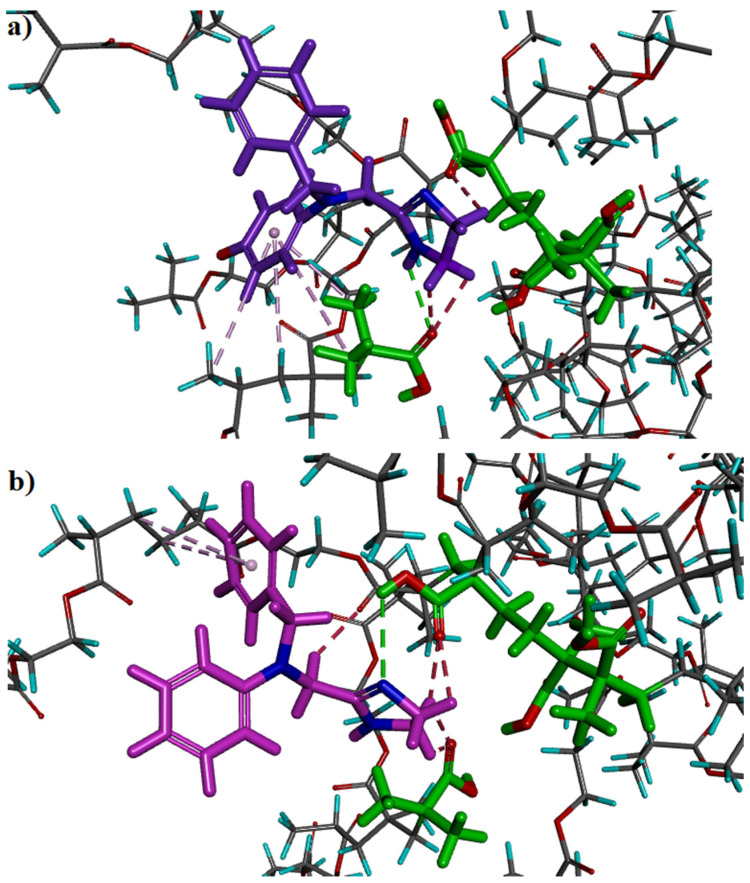
Views of ANT-O^–^ (**a**) and ANT (**b**) at the end of the adsorption process in the MIP model cavity (monomers—green color); classical hydrogen bonds—green dashed lines; non-classical hydrogen bonds—red dashed lines; hydrophobic interactions—light violet dashed lines.

The lowest Δ*E_B_* value (–92.5 kcal mol^−1^) was observed during analysis of the adsorption model of ANT-O^–^ in the MIP cavity. This fact could suggest that anionic form of ANT-O^–^ might effectively interact with MIP binding site. In this system, three types of interactions between ANT-O^–^ and the polymeric matrix were observed, namely, classical and non-classical hydrogen bonds as well as hydrophobic interactions (Figure 4a). The classical hydrogen bond was created between the H atom of –NH group (imidazole ring) from ANT-O^–^ and the O atom of –COOH group from the monomer residue (length of 2.57 Å). Three non-classical hydrogen bonds were formed between the H atoms of –CH_2_– groups (imidazole ring) from ANT-O^–^ and the O atoms of –COOH groups from two monomer residues (length between 2.51 and 2.98 Å). Finally, four hydrophobic interactions (π-alkyl type) between aromatic ring of ANT-O^–^ and –CH_2_– or –CH_3_ groups from monomer or EGDMA (ethylene glycol dimethacrylate) residues were observed (length between 3.92 and 5.42 Å).

The next model that was characterized by the negative value of Δ*E_B_* (–57.0 kcal mol^−1^), showed the adsorption process of ANT in the MIP cavity. In this system, the same types of interactions between the analyte and the MIP as in the ANT-O^–^–MIP model were observed (Figure 4b). The classical hydrogen bond was created between the N atom of imidazole ring from ANT and the H atom of –COOH group from the monomer residue (length of 3.09 Å). Five non-classical hydrogen bonds were formed between the H atoms of –CH_2_– groups (imidazole ring or alkyl chain) from ANT and the O atoms of –COOH groups from two monomer residues or an O atom from EGDMA residue (length between 2.33 and 2.55 Å). Finally, two hydrophobic interactions (π-alkyl type) between aromatic ring of ANT and –CH_2_– groups from EGDMA residues were observed (length between 5.10 and 5.19 Å).

**Figure A1:**
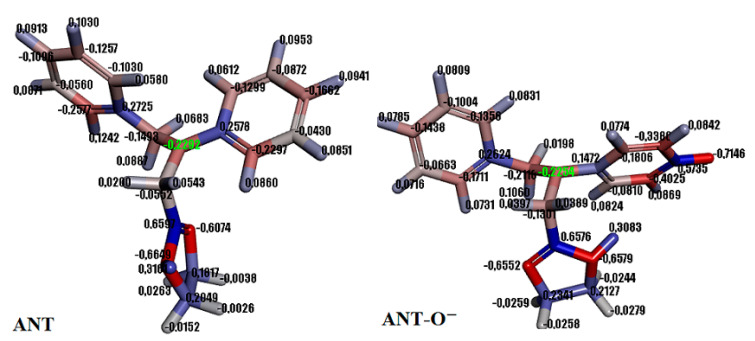
Distribution of atomic partial charges (ESP) in the molecules of ANT and ANT-O^–^. Molecules colored according to the partial charge values—negative values are shown as red and positive values as blue.

In the adsorption process of ANT and ANT-O^–^, the electrostatic interactions should be also taken into account. According to analysis of charge distribution in all studied structures, it was observed that both ANT and ANT-O^–^ had very similar values of charge on the N atom located in the alkyl chain: −0.2292 and −0.2254, respectively (Figure A1 in Appendix A). The charge values of the N atoms in alkyl chains in the ANT^+^, ANT-OH, or ANT^+^-O^–^ differed significantly from those observed for the ANT and ANT-O^–^ molecules. This fact could suggest that the N atom localized in the alkyl chain of studied analytes, strengthen the interactions of ANT or ANT-O^–^ with the MIP and could play a decisive role in the adsorption mechanism of ANT or ANT-O^–^ on the sorbent surface.

Next, the impact of solvent on the adsorption process was analyzed. The solvent could affect the strength of interactions between the analyte and polymer matrix. The adsorption attenuation could be related to the formation of interactions between the analyte and solvent molecules. Additionally, the solvent could enhance the non-specific adsorption on the MIP matrix. In both analyzed systems, viz. ANT–MIP and ANT-O^–^–MIP, interactions between analytes and solvent (methanol or water) molecules were observed. However, the higher number of solvent-analyte interactions was observed for ANT-O^–^–MIP system (five classical and two non-classical hydrogen bonds) when compared to the ANT–MIP (two classical and two non-classical hydrogen bond). This fact could be related to the presence of negatively charged O atom at the aromatic ring of ANT-O^–^. Both analytes created classical and non-classical hydrogen bonds between N atoms (from imidazole rings) or H atom (from –NH group of imidazole ring—only ANT) or O atom (only ANT-O^–^) or H atoms (from –CH_2_– groups of alkyl chains) from analytes and H or O atoms from methanol or water molecules. The interactions of analytes with solvent could weaken the adsorption process on the sorbent. It could be also emphasized that stronger interactions of ANT-O^–^ with the solvent could result in lower recovery values for this analyte observed in the SPE experiments.

#### 2.1.3. Morphology and Composition of Imprinted Sorbent

**Figure 5:**
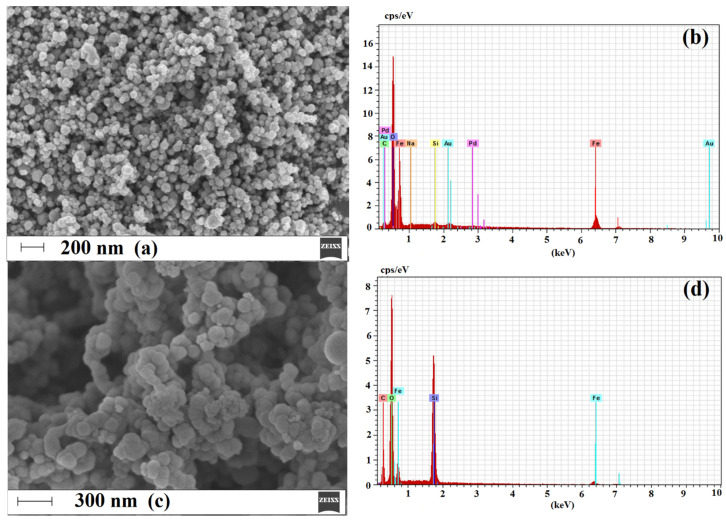

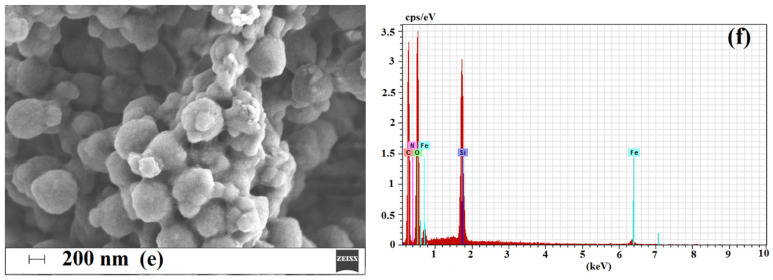
Micrographs of Fe_3_O_4_ (**a**), Fe_3_O_4_@SiO_2_-MPS (**c**) and mag-MIP (**e**) and EDS spectra of Fe_3_O_4_ (**b**), Fe_3_O_4_@SiO_2_-MPS (**d**) and mag-MIP (**f**).

Analysis of surface morphology is a very important step to prove the proceeding of subsequent layers on magnetite core. Thus, for such purpose, the field emission scanning electron microscopy (FE-SEM) was employed to investigate differences between neat Fe_3_O_4_ nanoparticles, Fe_3_O_4_ silanized and functionalized by MPS (3-(trimethoxysilyl)propyl methacrylate) (Fe_3_O_4_@SiO_2_-MPS) and final material, viz. mag-MIP (Fe_3_O_4_@SiO_2_-MPS@MIP). Additionally, the electron dispersive spectroscopy (EDS) analysis was used in order to confirm the structural composition of each subsequent layer. Figure 5 presents micrographs of Fe_3_O_4_, Fe_3_O_4_@SiO_2_-MPS and mag-MIP together with EDS analyses of corresponding layer. 

Micrograph of Fe_3_O_4_ revealed nanoparticles of quite uniform spherical shape (Figure 5a). The particles were agglomerated probably due to their magnetic nature. In contrast, the submicroparticles obtained after conjugation of silane layer functionalized with MPS were characterized by less uniform structure, containing agglomerates of spherical entities (Figure 5c). The external imprinted shell of mag-MIP was obtained after polymerization, and the surface of final material was characterized by numerous entities uniformly coated by the organic polymeric layer (Figure 5e).

The EDS analyses confirmed proceeding of each subsequent step by change in the elemental weight percentages (Figure 5b,d,f). The Fe_3_O_4_ nanoparticles were composed only from Fe and O with wt% values of 66.52 and 29.55, respectively. The composition of material after silanization and functionalization by MPS revealed the presence of the Si and C atoms as well as confirmed changes in the ratio of Fe and O. The following values of wt% for Si, C, Fe and O were obtained: 22.90, 16.19, 15.07, 45.83, respectively. The composition of mag-MIP revealed further changes in the ratio between the O and C atoms, proving conjugation of organic layer. The following values of wt% for Si, C, Fe and O were obtained: 17.55, 37.33, 9.14, 34.94, respectively. 

To sum up, the presented analyses confirmed obtaining of core–shell material and adsorption characteristics proved the presence of specific adsorption sites on the surface on mag-MIP with the respect of mag-NIP.

### 2.2. Optimization of Dispersive Solid Phase Extraction

In the optimization of d-SPE following variables were tested: the contact time during loading and elution of analyte, the type of eluent and the washing solvent. 

**Figure A2:**
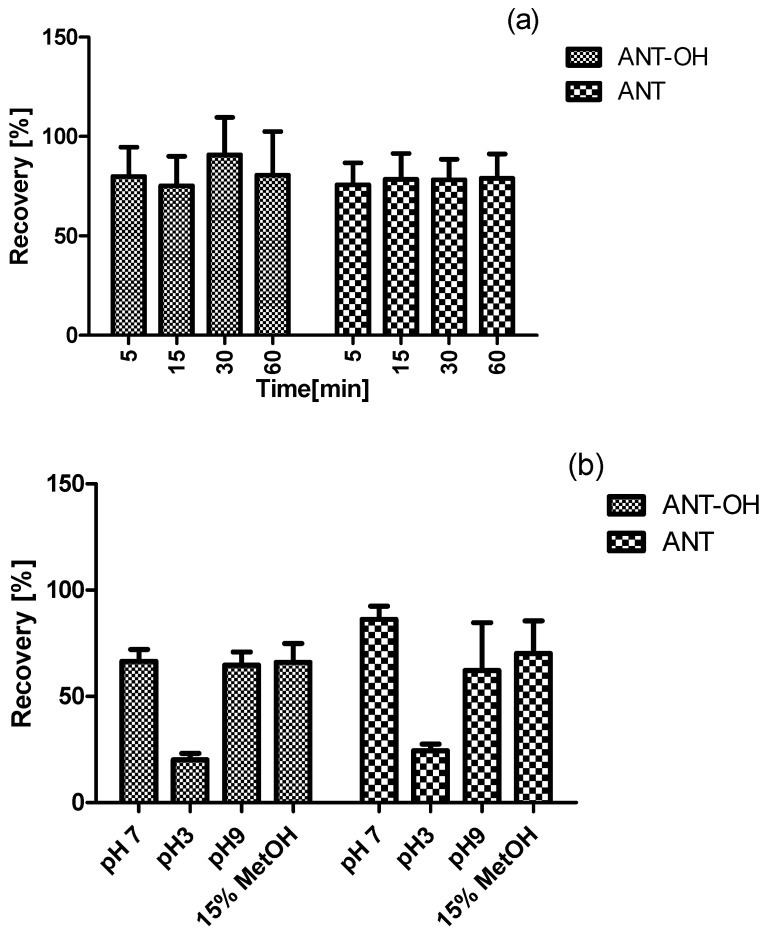

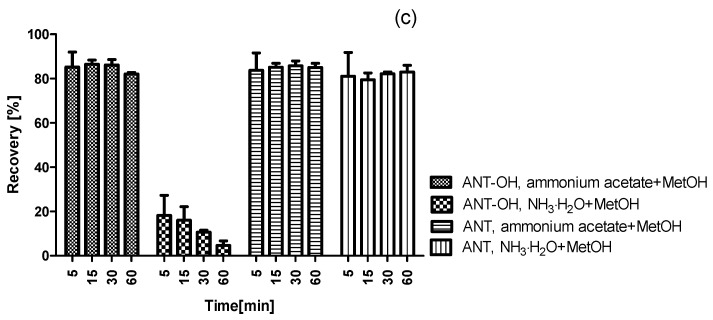
Results of optimization process of antazoline (ANT) and hydroxyantazoline (ANT-OH) extraction. Contact time of the analytes with the sorbent (**a**), type of washing solution (**b**) as well as time and type of elution solvent (**c**) were tested.

First the effect of the contact time of plasma time on method recovery was analyzed. Different times of contact were applied, viz. 5, 15, 30 or 60 min. As it could be seen (Figure A2a in Appendix A), recovery of ANT was very similar after 5, 15, 30 and 60 min of incubation, whereas the amount of adsorbed ANT-OH was significantly higher after 30 min comparing to other contact times. Thus, the 30 min loading time was selected as optimal. 

In the next step of optimization, the washing solvent was optimized. Different solvents were applied, viz. water, water adjusted to pH 3 and to pH 9, as well as 15% methanol (Figure A2b in Appendix A). For both analytes the lowest recoveries were noted for water adjusted to pH 3. The recoveries after application of other solvents were similar for ANT-OH. In the case of ANT, the recovery was the highest after application of water as washing solvent. Here, water was selected as optimal washing solvent.

In the following step the elution time and elution solvent were optimized. Equally high efficacy of elution was observed for all tested elution times. Similar effectiveness of extraction was noted for ANT for both tested elution solvents. In contrary, for 1% ammonium hydroxide in methanol ANT-OH recovery significantly decreased (Figure A2c in Appendix A). This result was proven by the theoretical analysis, confirming valuable predictions for the analyzed models. Thus, the 5 min elution time and 40 mM ammonium acetate-methanol (30:70, *v*/*v*) were selected as optimal. 

To sum up, the optimization of magnetic d-SPE allowed to propose efficient and facile protocol for extraction both ANT and ANT-OH form plasma. 

### 2.3. Validation of Method

The fragmentation pattern of ANT-OH was presented previously [15] and showed the fragment of *m*/*z* 191, 212, 161, 122, 91 and 71. Since ANT-OH was not determined previously using LC-MS, the optimization of MRM method was performed. The most abundant MRM transition was observed for *m*/*z* 282 > 91 (CE = 39 V) followed by *m*/*z* 282 > 212 (CE = 23 V). Thus, the *m*/*z* 282 > 91 was selected as the quantitative transition.

**Figure 6:**
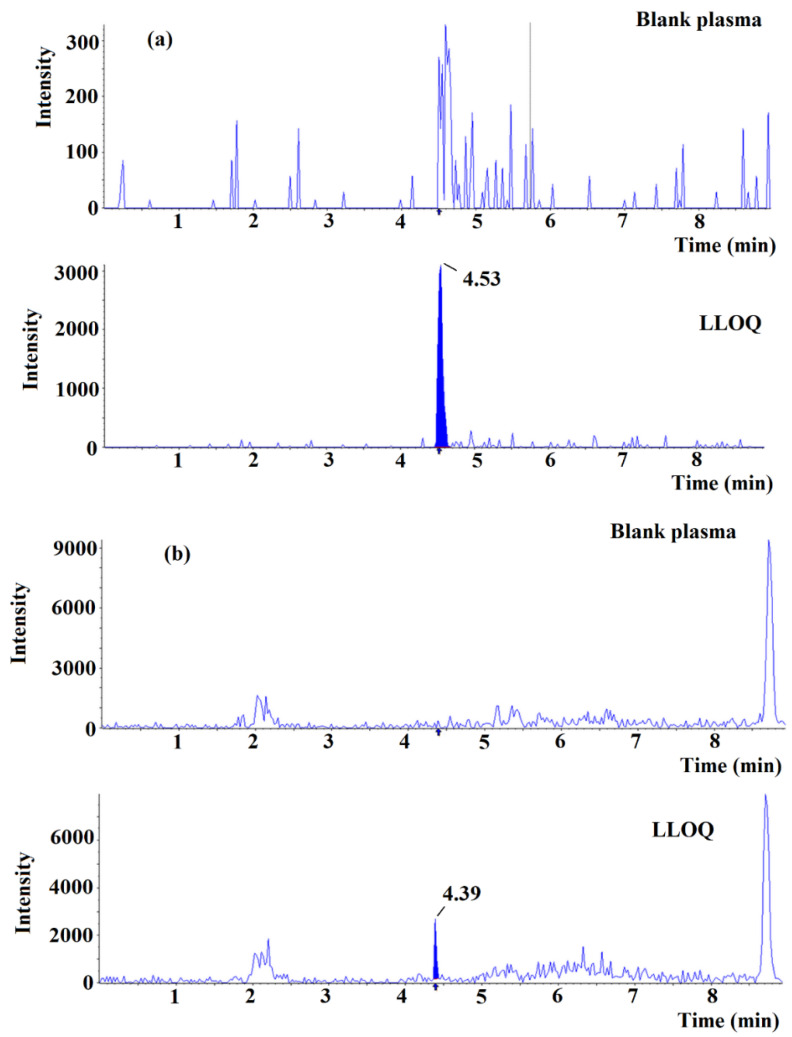
Extracted chromatogram of ANT (**a**) and ANT-OH (**b**) in blank plasma and lower limit of quantitation (LLOQ).

The calibration curve was obtained by the weighted quadratic regression analysis (x^−1^). The linearity range was over the concentration range of 3–500 µg L^−1^ for ANT-OH and 30–3000 µg L^−1^ for ANT. The values of regression parameters (and their standard deviation) described by the equation: y = ax + b, were as follows: a = 0.00136 ± 21, b = 0.0002 ± 12 for ANT-OH and a = 0.00138 ± 19, b = 0.0351 ± 96 for ANT with correlation coefficient r ≥ 0.99. The accuracy for lower limit of quantification (LLOQ) (Figure 6) of ANT-OH and ANT was 98% (RSD = 12%, *n* = 15) and 105% (RSD = 9.0%, *n* = 15) within three runs, and 90% (RSD = 10.0%, *n* = 5) and 103% (RSD = 9.9%, *n* = 5) within one day. The accuracy for the QC1 samples between runs was 98% (RSD 11%, *n* = 15) and 114% (RSD = 12%, *n* = 5), while within one day was 95% (RSD 11%, *n* = 5) and 100% (RSD 7.1%, *n* = 5) for ANT-OH and ANT, respectively. The inter-day accuracy for QC2 was 97% (RSD 10%, *n* = 15) for ANT-OH and 101% (RSD 5.5%, *n* = 15) for ANT, whereas for QC3 was 96% (RSD 9.0%, *n* = 15) for ANT-OH and 102% (RSD 5.2%, *n* = 15) for ANT. The intra-day accuracy for QC2 was 88% (RSD 4.9%, *n* = 5) and 98% (RSD 5.6%, *n* = 5), whereas for QC3 was 106% (RSD 3.2%, *n* = 5) and 100% (RSD 3.0%, *n* = 5) for ANT-OH and ANT, respectively. No carry-over was detected. The method was selective regarding ANT-OH, ANT and internal standards. 

The matrix factor calculated for QC1 was 79% for ANT-OH and 91% for ANT, whereas for QC3 was 68% for ANT-OH and 93% for ANT. The variation of relative matrix factor was 14% (ANT-OH) and 12% (ANT) for QC1, and 12% (ANT-OH) and 4.7% (ANT) for QC3. The recovery for QC1 was 88% (RSD 11%) for ANT-OH and 68% (RSD 8.0%) for ANT, whereas for QC3 was 77% (RSD 6.8%) for ANT-OH and 80% (RSD 2.0%) for ANT. The selectivity of imprinted materials is revealed predominantly in lower concentrations. In contrary, higher concentrations of analytes could be responsible for increase of non-specific adsorption. This phenomenon could explain nearly similar percentage of recoveries of ANT-OH and ANT for QC3. The recovery of ANT for high quality control (the only one reported) was slightly higher in d-SPE method (80%, RSD = 2.0%) than in previously reported methods based on cloud point extraction (70%, RSD = 5.3%) and LLE (65% RSD = 6.6%) [18].

Processed samples were stable up to 24 h in the autosampler (ANT: 98–100%, ANT-OH: 95–105%). 

To summarize, all tested validation criteria were fulfilled.

### 2.4. Pharmacokinetics

**Figure 7:**
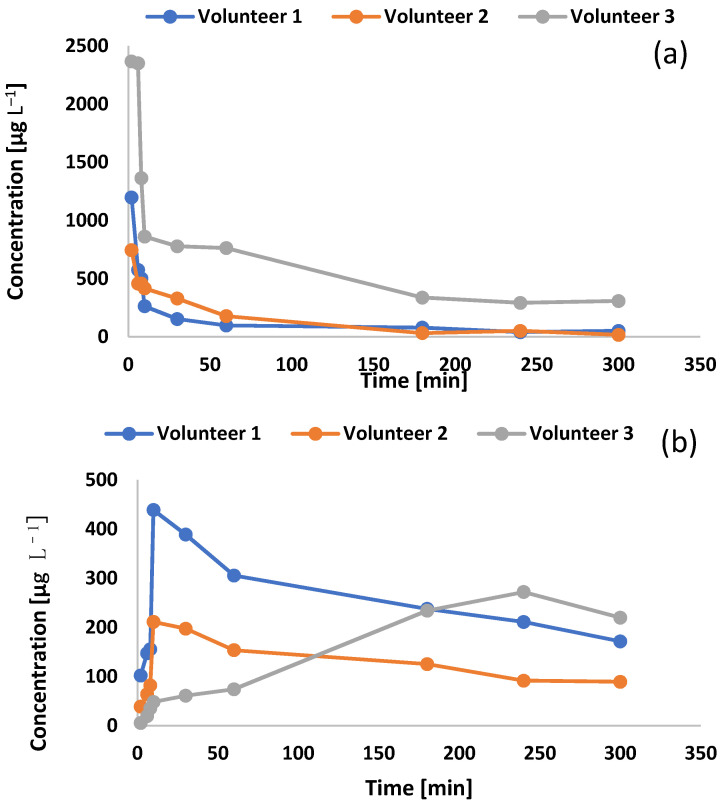
Pharmacokinetic profile of (**a**) antazoline (ANT) and its metabolite (**b**) hydroxyantazoline (ANT-OH) after intravenous administration of 100 mg antazoline mesylate to three volunteers.

The plasma concentration-time profile for ANT-OH and ANT for volunteers are presented in Figure 7. The highest concentration of ANT-OH in plasma was observed after 10 min for two volunteers and after 240 min for the third one. Significant differences between the first two and the third volunteer could be explained by slower ANT metabolism. As presented in Figure 7, ANT concentration decreased slower than in the case of the first two volunteers. Moreover, the concentration of the parent drug at 300 min post dose was around LLOQ (30 µg L^−1^) for the first two volunteers, whereas for the third one was 220 µg L^−1^. The possible explanation can be polymorphism of CYP2D6, gene with more than 100 allelic variants. As we have shown recently, the cytochrome is the main CYP isoform involved in the metabolism of ANT [15]. Thus, the velocity of ANT metabolism can be influenced by the CYP2D6 activity. The prevalence of poor metabolizers in Europe varies from 15.7% in Finland and Turkey to 33.6% at Faroe Islands [27]. However, to confirm the hypothesis, the genetic test is needed. The maximum ANT-OH concentration was 307 ± 96 µg L^−1^. The elimination half-life was 4.39 ± 0.55 h. Area under curve of plasma concentration until the last concentration observed was 930 ± 270 h µg L^−1^. The pharmacokinetic of ANT was presented previously [14].

## 3. Materials and Methods

### 3.1. Chemicals

The template molecule of 2-(4-imidazolyl)ethylamine dihydrochloride, the monomer of methacrylic acid and cross-linker of EGDMA, tetraethoxysilane, MPS and 2,2′-azobis(2-methylpropionamidine) dihydrochloride, the initiator, were purchased from Sigma-Aldrich (St. Louis, MO, USA). Trisodium citrate dehydrate, sodium hydroxide, sodium nitrate, ferrous sulphate heptahydrate, ammonium hydroxide, ammonium acetate, methanol, ethanol, toluene and acetone were delivered from POCH (Gliwice, Poland). Ultrapure water was delivered from a Hydrolab HLP 5 system (Straszyn, Poland).

The reference standard of antazoline hydrochloride and xylometazoline (2-[(4-tert-butylo-2,6-dimetylofenylo)metylo]-4,5-dihydro-1H-imidazol, an internal standard ) were purchased from Toronto Research Chemicals (Toronto, ON, Canada). Oxymetazoline (6-tert-butylo-3-(4,5-dihydro-1H-imidazol-2-ilometylo)-2,4-dimetylofenol, an internal standard) were purchased from Sigma-Aldrich (St. Louis, MO, USA). The solvents, HPLC gradient-grade methanol, acetonitrile and formic acid 98% were purchased from Merck (Darmstadt, Germany). Blank plasma was obtained from the Regional Centre of Blood Donation and Treatment (Warsaw, Poland).

### 3.2. Sorbent

The magnetic core was prepared as described previously [28] prior to the functionalization by a silane derivative, providing the functional groups and enabling the polymerization of imprinted layer on its surface. Details of the synthesis of magnetic core and functionalization can be found in Appendix A.1.

The magnetic core–shell polymerization process was proceeded similarly to previously described [29] to prepare imprinted Fe_3_O_4_@SiO_2_-MPS@MIP (coded as mag-MIP) and non-imprinted Fe_3_O_4_@SiO_2_-MPS@NIP (coded as mag-NIP) polymers. For synthesis of mag-MIP, the structural analog of target analyte was used as the template [30] and for synthesis of mag-NIP the addition of template was omitted. Briefly, to a volume of 25 mL of methanol, 36.8 mg (0.2 mmol) of 2-(4-imidazolyl)ethylamine dihydrochloride and 68.9 mg (0.8 mmol) of methacrylic acid were added, followed by addition of a volume of 754 µL (4 mmol) of EGDMA, 20 mg of 2,2′-azobis(2-methylpropionamidine) dihydrochloride and 215.4 mg of Fe_3_O_4_@SiO_2_-MPS. The mixture was sonicated for 5 min, purged with nitrogen for 5 min and left heated to 60 °C on the magnetic stirrer overnight for proceed polymerization process. Next, the polymer was separated and washed (using external magnet) in a following sequence: methanol (2 × 20 mL), 40 mM aqueous ammonium acetate–methanol 30:70 *v*/*v* (2 × 20 mL), and methanol (2 × 20 mL). For mag-MIP, template removal step was proceeded in the Soxhlet apparatus, lasting 36 h (120 mL of methanol) and was monitored by LC-MS.

The surface morphology analysis using scanning electron microscopy (SEM) with a Merlin FE-SEM (Zeiss, Germany) and EDS analysis using an EDS X-ray detector (Brucker, Germany) were performed at the Faculty of Chemistry, University of Warsaw, Poland. The samples were Au/Pd sputter-coated before SEM analysis. 

### 3.3. Instruments

Quantitative analysis was performed using an Agilent 1260 Infinity system (Agilent Technologies, Santa Clara, CA, USA), equipped with a degasser, an autosampler and a binary pump coupled to QTRAP 4000 hybrid triple quadrupole/linear ion trap mass spectrometer (AB Sciex, Framingham, MA, USA). The turbo ion spray source was operated in positive mode. The curtain gas, ion source gas 1, ion source gas 2 and collision gas (all high purity nitrogen) were set at 345 kPa, 207 kPa, 276 kPa and “high” instrument units (4.6 × 10^−5^ Torr) respectively. The ion spray voltage and source temperature were 5000 V and 600 °C, respectively. The target compounds were analyzed in multiple reaction monitoring (MRM) mode. The quantitative MRM transitions, declustering potential (DP) and collision energy (CE) was *m*/*z* 282 > 91 (DP = 96 V, CE = 39 V) for ANT-OH, *m*/*z* 266 > 65 (DP = 81 V, CE = 25 V) for ANT, *m*/*z* 245 > 145 (DP = 121 V, CE = 63 V) for xylometazoline, *m*/*z* 261 > 57 (DP = 111 V, CE = 49 V) for oxymetazoline. Chromatographic separation was achieved with a Kinetex^®^ C18 column (100 mm × 4.6 mm, 2.6 µm) from Phenomenex (Torrance, CA, USA). The column was maintained at 40 °C at the flow rate of 0.5 mL min^−1^. The mobile phases consisted of 0.2% formic acid as eluent A and acetonitrile with 0.2% formic acid as eluent B. The gradient (%B) was as follows: 0 min 20%, 1 min 20%, 3 min 95% and 6 min 95%. The re-equilibration of the column to the initial conditions lasted 3 min. 

### 3.4. Binding Study and Sample Preparation

For isotherm analysis, the polypropylene tubes were filled with 5 mg of mag-MIP or mag-NIP particles and a volume of 1 mL of different methanol-water (85:15 *v*/*v*) standard solutions of ANT and ANT-OH (concentrations between 0.5–50 μg L^−1^, extended to 250 μg L^−1^ for ANT-OH) were added. The tubes were sealed and oscillated by a shaker at room temperature for 45 min. Then, the tubes were centrifuged and the aliquots of supernatant were used to analyze the unbound amounts of each compound by LC-MS. For kinetics, the tubes were prepared as above but different times of oscillation were employed (15, 30, 45, 90, 120 and 180 min). Then, the tubes were treated in the same manner as described above. All measurements were carried out in triplicate. The binding capacities (*B*, μmol g^−1^) of mag-MIP or mag-NIP were calculated according to Equation (1):

*B* = (*C_i_* − *C_f_*)*V*/*M* (1)

where, *V* represents a volume of solution (L), *C_i_* represents the initial solution concentration (μmol L^−1^), *C_f_* represents the solution concentration after adsorption (μmol L^−^^1^) and *M* is the mass of particles (g). The adsorption isotherms for ANT and ANT-OH were characterized using the Freundlich model presented in Equation (2):

*B* = *aF^m^* (2)

where, *a* is the measure of the capacity (*B_max_*), *m* is a heterogeneity index and *F* is the concentration of the analyte in equilibrium state. For ANT-OH, Langmuir model transformed into the Scatchard equation was employed, according to Equation (3):

*B*/*F* = (*B_max_* − *B*)/*K_d_* (3)

where, *K_d_* is the dissociation constant. 

The kinetics of adsorption of ANT and ANT-OH were calculated using Ho-McKay model, according to Equation (4):

*t*/*q_t_* = (1/*k*_2_*q_e_*^2^) + (1/*q_e_*)*t* (4)

where, *k*_2_ is the second-order-rate constant at the equilibrium (g µg^−1^ min^−1^), *q_e_* is the adsorption capacity at equilibrium (µg g^−1^), *q_t_* is the adsorption capacity at *t*, time (in min), and using Weber-Morris model, according to Equation (5):

*q_t_* = *k*_3_*t*^1/2^ + *I* (5)

where *k*_3_ is the intra-particle diffusion rate constant (µg g^−1^ min^−1/2^) and *I* is a boundary layer thickness (µg g^−1^).

For the optimization of d-SPE process, a volume of 990 µL of untreated plasma sample (5000 μg L^−1^ of ANT and ANT-OH) and 10 µL of internal standards (c = 1000 μg L^−1^) were mixed and a volume of 300 µL of fortified plasma was transferred to Eppendorf tube with 5 mg of mag-MIP. Next, the tube was put on vortex to provide a contact time with sorbent for 30 min (in the optimization of loading time, the step lasted 5, 15, 30 or 60 min). Then, the supernatant, separated from the sorbent by an external magnetic field, was discarded and the washing step was carried out by applying a volume of 300 μL of ultra-pure water for 0.5 min on the vortex (or in the optimization process: water adjusted to pH 9 (with ammonium hydroxide), water adjusted to pH 3 (with formic acid), methanol-water 15:85 *v*/*v*). The supernatant was removed in the same manner as it was described above. Finally, the elution took place by adding a volume of 1000 µL of 40 mM ammonium acetate-methanol, 30:70 *v*/*v* (or in the optimization process 5% ammonium hydroxide in methanol). The elution time was set for 5 min (in the optimization process the step lasted 5, 15, 30 or 60 min). The elution fraction was separated from sorbent by the application of external magnetic field. In each optimized step the recoveries of ANT and ANT-OH were analyzed. The eluate was diluted with water pH 3 (1:1 *v*/*v*) and an aliquot of 10 µL was injected into the LC-MS. Each experiment was performed in triplicate.

### 3.5. Method Validation

The method was validated according to European Medicines Agency (EMA) guideline [31]. The following parameters were analyzed: LLOQ, calibration curve performance, precision, accuracy, matrix effect, recovery, selectivity, carry-over and stability of the analyte in the extract. Calibration curves (*n* = 6) for both analytes were constructed with eight calibration points. The LLOQ for the analytes were determined as the lowest concentration of the calibration curves and analyzed for precision, accuracy and signal to noise ratio. The accuracy and precision of the method were determined within run (*n* = 5) and between run (*n* = 15), using LLOQ (3 µg L^−1^ for ANT-OH and 30 µg L^−1^ for ANT) and QC samples (9, 250 and 375 µg L^−1^ for ANT-OH and 90, 1500 and 2250 µg L^−1^ for ANT). Carry-over wherein blank human plasma samples were analyzed following the highest calibration standards was also studied. Matrix effect and recovery were evaluated at QC1 and QC3 concentrations with six lots of plasma including plasma with haemolysis and plasma with lipemia. The matrix effect was assessed by comparing the peak area of samples spiked after extraction with that of the standard solution containing equivalent amount of the analytes. The recovery was calculated by comparing the peak area of samples spiked before and after extraction. The method selectivity was analyzed using six lots of plasma. The autosampler stability of the analytes in extract was determined at two concentration levels (QC1 and QC3) directly after sample preparation and 24 h after storage in an autosampler (4 ± 0.5 °C).

### 3.6. Pharmacokinetic Study

The study protocol was approved by the Local Ethics Committee of the Postgraduate Medical School, Warsaw, Poland (17/PB/2014, 26 March 2014). Three healthy volunteers (men, age 25–30 years) were enrolled in this study after obtaining a written informed consent. 

All participants were non-smokers with body weight no less than 50 kg and body mass index ranging from 19 to 24. Subjects neither consumed alcohol nor received any medication within 2 weeks before the start of the study. The exclusion criteria were lack of written informed consent, younger than 18 years, allergic to antazoline, presence of significant heart disease such as heart failure with NYHA class >II or left ventricular ejection fraction (LVEF) < 40%, uncontrolled hypertension or documented coronary artery disease. Other contraindications included cardiac rhythm other than sinus, systolic blood pressure (SBP) < 90 mmHg, presence of liver or kidney failure or/and chronic obstructive pulmonary disease or/and diabetes mellitus, active inflammatory process.

Antazoline mesylate was given intravenously in 100 mg bolus injected during 1 min. An approximate volume of 2 mL of blood samples were collected (to vials containing sodium citrate as an anticoagulant, 3.2%) before and at 2, 6, 10, 30, 60, 180, 240 and 300 min post-dose. After collection, blood samples were centrifuged at 2000 *g* for 15 min at room temperature and plasma samples were analyzed. The pharmacokinetic parameters were calculated using non-compartmental analysis tool of PKSolver, a freely available menu-driven add-in program for Microsoft Excel written in Visual Basic for Applications (VBA) [32]. The area under the plasma concentration versus time curve (AUC) was calculated from 0 to 300 min by the linear trapezoidal method. The apparent terminal elimination rate constant, λz, was obtained by linear regression of the log-linear terminal phase of the concentration-time profile by using at least three non-zero declining concentrations in terminal phase with a correlation coefficient of >0.8. The terminal half-life value (t_1/2_) was calculated using the equation (ln2) × λz.

### 3.7. Theory

The geometries of molecules were optimized using density functional theory with a B3LYP/6-311+G(d,p) hybrid functional implemented in the Gaussian 09 software [33]. The so-called ESP (electrostatic potential) atomic partial charges on the atoms were computed using the Breneman model [34], reproducing the molecular electrostatic potential. To build the models of analyzed systems, the Packmol software [35] was used. Packmol enables creating the starting conformations of the systems for all simulations, and packs molecules randomly into defined points of the simulations area. Molecular mechanics (MM) and molecular dynamics (MD) simulations were run using the CHARMM force field [36] implemented in the appropriate module of BIOVIA Discovery Studio 2019 [37]. The MM process consisted of 100 steps of steepest descent and then 10,000 steps of conjugate gradient minimization cycles. The process was applied until the RMS (root-mean-square) gradient of the structure fell below 0.01 kcal mol^−1^ Å^−1^. The MD simulation process contained a heating step (from 0 to 300 K) performed for 100 ps with time steps of 1 fs. In the next step, isothermal equilibration was performed for 100 ps at 300 K. The Leapfrog Verlet integration and SHAKE [38] algorithms were used during the simulation process. The production run was conducted for 5 ns in the NVT ensemble (constant-volume/constant-temperature dynamics) at 300 K and the coordinates were recorded every 10 ps. Trajectory file data generated from the NVT MD simulation have been used in all the calculations and analyses presented in this research.

Theoretical designing process of MIP cavity model consisted of two steps. First, the prepolymerization complex model was created. The box with the template—histamine molecule surrounded by 4 methacrylic acid molecules (functional monomers)—was constructed and then 20 molecules of EGDMA (cross-linker) and 309 molecules of methanol (porogen) were added. Starting structure for MD simulation was obtained using Packmol. Next, the MM and MD calculations were performed and the model of prepolymerization complex was constructed. The molecules’ number of system components was chosen to mimic their molar ratio used during the synthetic procedure. In the next stage of polymer modeling, the creation of polymeric chain with the binding site was performed. The optimized structure of prepolymerization complex with the minimum potential energy value was chosen for further investigation. Single bonds between vinyl groups of methacrylic acid and EGDMA molecules in the prepolymerization system were created. Bonds were formed between the nearest vinyl C atoms of monomer or cross-linker molecules but taking into account the fact that all molecules of monomer and cross-linker in studied system should create one cross-linked polymeric chain. Then, the H atoms were added to the chain structure. The latter protocol mimicked the polymerization reaction during the synthesis of MIP. So-called ESP charges were calculated for created structure of polymer chain. Next, the MM and MD procedures were repeated for systems constructed on the basis of prepolymerization complex model and consisted of polymeric chain, the template and solvent to form specific binding site model in the polymeric matrix. 

The final stage of computational analysis involved simulations of adsorption process of chosen analytes: ANT and ANT-OH. The optimized structure of polymer chain with binding site model with the minimum potential energy value was chosen for further investigations. The template was removed to create the MIP cavity and the empty space was proposed as the computer model of binding site. Then, the molecules of chosen analytes, ANT or ANT-OH in different forms: neutral (ANT, ANT-OH), cationic (ANT^+^), anionic (ANT-O^–^) or zwitterionic (ANT^+^-O^–^), were inserted into the model of MIP cavity (replacing the template molecule). The solvent, consisting of 418 molecules of methanol and 169 molecules of water, was added and starting structures for MD procedure were formed with the use of Packmol. Next, the MD simulations were carried out, mimicking the experimental conditions of adsorption.

The binding energies (Δ*E_B_*, kcal mol^−1^) were calculated according to Equation (6):

Δ*E_B_* = *E_system_* − *E_analyte_* − *E_cavity_* (6)

where, *E_system_* is the potential energy of the MIP cavity with bound analyte in the solvent, *E_analyte_* is the potential energy of appropriate analyte and *E_cavity_* is the potential energy of the MIP cavity without the analyte in the solvent.

## 4. Conclusions

Magnetic core–shell imprinted nano-conjugates allowed for efficient separation of antazoline and hydroxyantazoline from human plasma. The adsorption data confirmed heterogeneous population of binding sites towards hydroxyantazoline. The adsorption properties were interpreted by the theoretical studies. Optimization of magnetic dispersive solid phase extraction provided a fast, facile and reliable pretreatment step. The SPE recoveries were proved by the theory, confirming valuable predictions for the analyzed models. The proposed analytical method is the first one to determine the antazoline metabolite concentration. Validation experiments proved that the developed method is accurate, precise and specific. The method was applied for pharmacokinetic studies of intravenous administration of 100 mg antazoline mesylate. Significant intersubject variability in pharmacokinetic profile of antazoline and its metabolite were noted. Further experiments are needed to confirm whether difference is caused by the CYP2D6 polymorphism.

## Data Availability

The data presented in this study are available on request from the corresponding author.

## Appendix A

### Appendix A.1. Synthesis of Magnetic Core and Functionalization

The Fe_3_O_4_ magnetic nanoparticles were synthetized by addition of 2.94 g (10 mmol) of trisodium citrate dehydrate, 1.63 g (40 mmol) of sodium hydroxide and 34.2 g (400 mmol) of sodium nitrate which were dissolved in a volume of 180 mL of ultrapure water. The reaction was carried out in a round-bottom flask equipped with reflux and placed on a stirrer with heating plate (Heidolph, Germany). The mixture was stirred and heated to 100 °C until a clear solution was observed. A volume of 20 mL of 1 mol L^−1^ aqueous ferrous sulphate heptahydrate solution was added and was maintained at 100 °C for one hour (h). After letting it cool down slowly to room temperature, a dark brown precipitate was observed. It was separated by a magnet (discarding the solution) and then the particles were washed with ultrapure water for several times (20 mL, each). Then, the obtained Fe_3_O_4_ magnetic nanoparticles were dried at room temperature prior to the modification with tetraethoxysilane to obtain Fe_3_O_4_@SiO_2_. An amount of 600 mg of Fe_3_O_4_ was suspended in a volume of 80 mL of ethanol and 8 mL of ultrapure water under ultrasonication (Bandelin, Germany) for 15 min (min) in a round bottom flask. After that, a volume of 10 mL of ammonium hydroxide and a volume of 4 mL of tetraethoxysilane were added. The reaction was carried out for 12 h at room temperature with stirring. The product was separated with a magnet and was washed with ultrapure water and ethanol (20 mL, each). Afterwards, the reaction of the Fe_3_O_4_@SiO_2_ with MPS was carried out to provide Fe_3_O_4_@SiO_2_-MPS. An amount of 500 mg of the Fe_3_O_4_@SiO_2_ was suspended in a volume of 100 mL of anhydrous toluene and 10 mL of MPS in a three-neck round bottom flask. The mixture was allowed to react for 12 h under nitrogen. The Fe_3_O_4_@SiO_2_-MPS was separated again with a magnet and washed with ultrapure water (20 mL, each). The prepared particles were left in dry conditions for further synthesis of molecularly imprinted shell.
